# Root Transcriptome Analysis of Wild Peanut Reveals Candidate Genes for Nematode Resistance

**DOI:** 10.1371/journal.pone.0140937

**Published:** 2015-10-21

**Authors:** Patricia M. Guimaraes, Larissa A. Guimaraes, Carolina V. Morgante, Orzenil B. Silva, Ana Claudia G. Araujo, Andressa C. Q. Martins, Mario A. P. Saraiva, Thais N. Oliveira, Roberto C. Togawa, Soraya C. M. Leal-Bertioli, David J. Bertioli, Ana Cristina M. Brasileiro

**Affiliations:** 1 EMBRAPA Genetic Resources and Biotechnology, Brasilia, DF, Brazil; 2 EMBRAPA Semi–Arid, Petrolina, PE, Brazil; 3 University of Brasilia, Brasilia, DF, Brazil; Louisiana State University Agricultural Center, UNITED STATES

## Abstract

Wild peanut relatives (*Arachis* spp.) are genetically diverse and were adapted to a range of environments during the evolution course, constituting an important source of allele diversity for resistance to biotic and abiotic stresses. The wild diploid *A*. *stenosperma* harbors high levels of resistance to a variety of pathogens, including the root-knot nematode (RKN) *Meloidogyne arenaria*, through the onset of the Hypersensitive Response (HR). In order to identify genes and regulators triggering this defense response, a comprehensive root transcriptome analysis during the first stages of this incompatible interaction was conducted using Illumina Hi-Seq. Overall, eight cDNA libraries were produced generating 28.2 GB, which were *de novo* assembled into 44,132 contigs and 37,882 loci. Differentially expressed genes (DEGs) were identified and clustered according to their expression profile, with the majority being downregulated at 6 DAI, which coincides with the onset of the HR. Amongst these DEGs, 27 were selected for further qRT-PCR validation allowing the identification of nematode-responsive candidate genes that are putatively related to the resistance response. Those candidates are engaged in the salycilic (NBS-LRR, lipocalins, resveratrol synthase) and jasmonic (patatin, allene oxidase cyclase) acids pathways, and also related to hormonal balance (auxin responsive protein, GH3) and cellular plasticity and signaling (tetraspanin, integrin, expansin), with some of them showing contrasting expression behavior between Arachis RKN-resistant and susceptible genotypes. As these candidate genes activate different defensive signaling systems, the genetic (HR) and the induced resistance (IR), their pyramidding in one genotype via molecular breeding or transgenic strategy might contribute to a more durable resistance, thus improving the long-term control of RKN in peanut.

## Introduction

Peanut (*Arachis hypogaea*) is the fourth most important oil crop in the world and is a major source of edible oil and digestible protein, especially in Asia, Africa and Americas. This crop also has a significant role in sustainable agriculture in terms of global food security and nutrition, fuel and energy, sustainable fertilization, and enhanced agricultural productivity as a rotation crop [1]. The narrow genetic base of the cultivated genepool together with the tetraploid and complex nature of the genome are a challenge to breeding efforts to develop cultivars with multiple resistances, high quality and yield [2]. Diversely, peanut diploid wild relatives (*Arachis* spp.) are important sources of resistance genes due to their higher genetic diversity, and because they have been selected during evolution in a range of environments and biotic stresses, constituting a rich source of new alleles to be introgressed into the cultivated species [3].

Peanut yield is worldwide affected by fungi, bacteria, virus and the root-knot nematode (RKN) *Meloidogyne arenaria*, which causes substantial yield losses at relatively low population densities [4]. The current approach to manage *M*. *arenaria* in the crop include chemical control, adequate cultural practices and most recently, the use of resistant cultivars, which once integrated with good pest management practices, can reduce the risk of pest outbreaks and the expense and environmental toxicity of nematicides [5]. Up to now, three peanut nematode resistant cultivars (COAN, Nematan and Tifguard) have been developed, all by the introgression of a chromosome region from the wild diploid species *A*. *cardenasii* [6, 7], for which recently, tightly linked genetic markers have been developed [4, 8]. Whilst quantitative sources of resistance to RKN have been found in peanut, strong resistance probably derived from a small number of genes, such as displayed by Hypersensitive Reaction (HR), has only been identified in diploid species [9, 10]. Therefore, the identification of novel genes related to plant defense against RKN in wild *Arachis* species is desirable, to broaden the sources of resistances deployed in peanut, and also secure greater durability of the resistance.

Resistance to RKN in various species is often mediated by HR, which occurs soon after invasion and is characterized by the generation of reactive oxygen species (ROS) in plant root cells associated with the presence of second stage juveniles (J2), with the *Mi-1*-mediated resistance in tomato being the most well characterized [11]. In previous studies [10], we showed that the wild peanut relative *A*. *stenosperma* is resistant to *M*. *arenaria*, with its mechanism of resistance operating in at least two stages: firstly, only few nematodes intrude the resistant roots, and then, those that successfully penetrated, were killed by HR in the surrounding cells. Transcriptional profiling studies in this species have identified a number of genes potentially related to this defense response [12–14]. Nevertheless, the molecular events and the functional role of these genes are yet to be clarified. In this regard, high-throughput mRNA sequencing (RNA-Seq) can be a complementary strategy to predict the roles and interactions of genes, and help the elucidation of complex signaling pathways activated in response to nematode parasitism. This technique enables a comprehensive transcriptome survey of induced changes in gene expression, and has already been successfully applied for the identification of genes responsive to diverse biotic and abiotic stresses, or during specific developmental stages in peanut [2, 15–17].

In this work, a genome-wide overview of gene expression during the first stages of the incompatible *A*. *stenosperma*/*M*. *arenaria* interaction was obtained using RNA-Seq, aiming to better understand the ways in which the resistant host transcriptome respond to the attack of this endoparasite and identify novel genes potentially involved with this nematode resistance. This approach provided new insights into the defense mechanisms used by this species to avoid infection and the ultimate development of the HR response. The understanding of these mechanisms and genes underlying this response will facilitate their transference to more adapted cultivars through the use of tightly linked genetic markers or via plant transformation, leading to improved peanut varieties with higher and more durable resistance to RKN.

## Material and Methods

### Plant material and libraries construction

*A*. *stenosperma* (accession V10309) plants challenged with *M*. *arenaria* race 1 were obtained as described before [14]. Shortly, plants were maintained under greenhouse conditions at the University of California, Riverside, USA, and approximately 20,000 juveniles of *M*. *arenaria* (J2) were resuspended in 1 ml of fresh deionised water and pipetted into soil depressions around each root of the 4-week-old plants. Control roots were collected at day zero and inoculated roots were collected at 3, 6 and 9 days after inoculation (DAI) in liquid nitrogen for total RNA extraction, using the lithium chloride modified protocol [18], and then purified using Invisorb Plant RNA Mini Kit (Invitek, Germany). Equal amounts of total RNA per collecting point (control and 3, 6 and 9 DAI) were pooled from five different plants, forming four combined samples with two independent biological replicates each. For the paired-end cDNA libraries construction and sequencing, services of FASTERIS (www.fasteris.com) were used employing the mRNA-seq and TruSeq (TM) SBS v5 protocols (Illumina, USA) on a Hi-Seq 2000 sequencing system.

### Sequence processing and analysis

The *de novo* contig assembly of *A*. *stenosperma* reads was performed by FASTERIS using Velvet assembler (http://www.ebi.ac.uk/~zerbino/velvet/) that takes in short read sequences removes errors and produces high quality unique contigs, followed by its additional module specialized in transcriptome assembly, OASES, which allows ambiguity assembly, such as alternative splicing or polymorphism (http://www.ebi.ac.uk/~zerbino/oases/). To validate the *de novo* assembly and estimate the number of assembled reads, a BWA mapping (http://bio-bwa.sourceforge.net/) was carried on with the *de novo* contigs for the best assembly selected, using 1 million pairs of each library. A complete mapping was only carried for the best selected assembly. The coverage of each transcripts of the best oases assembly was computed using the BEDtools software (http://code.google.com/p/bedtools).

Differential expression analysis of contigs was performed with DESeq package (http://www.bioconductor.org/biocLite.R) using the contig count matrix built from the Bedtools coverage output. Scaling factor for each given sample in each performed pairwise treatment-control was computed using the “estimateSizeFactors” function. Dispersion estimate call was performed with the “estimateDispersions” function from DESeq package for null model evaluation with few replicates with parameters method = "pooled", fitType = "parametric" and sharingMode = "fit-only". The read counts for each contig in each treatment-control condition were fitted to a negative binomial (NB) distribution via the “nbinomTest”- algorithm that involves borrowing information from count data for the complete set of genes. Transcript abundance for each gene was then inferred from the NB mean, in combination with a normalisation obtained by matching the distribution of read counts over a subset of genes which are likely not to be Differentially Expressed Genes (DEGs) according to the model fit. Thus, the null hypothesis corresponding to no differential expression is that the transcript abundance is the same in both conditions. P-values for each estimate of False Discovery Rates (FDRs) were added using the Benjamini-Hochberg algorithm.

For each time point (3, 6 and 9 DAI), the number of DEGs in the infected libraries was compared to the control samples to estimate the Fold Change (FC). Genes were considered as significant DEGs when their relative gene expression levels showed at least 4-FC (log2FC ≥2.0 or ≤ -2.0) difference between inoculated and control samples, with P-value<0.05. Clustering analysis of the significant DEGs based on common expression patterns was conducted using the Multiexperiment Viewer (MeV) (http://www.tm4.org/mev.html). K-means clustering was performed using TM4: MeV 4.7 software (http://www.tm4.org/mev.html) and Pearson’s correlation coefficient.

### Similarity search and functional annotation

Functional annotation of contigs was performed by sequence similarity searches using BLASTX against NCBI’s non-redundant sequence database, and similarities were considered significant with E-values ≤1e-7 (S1 Table). InterProScan (http://www.ebi.ac.uk/Tools/InterProScan/) was employed to perform protein domain and motif searches. Gene ontology (GO) terms were assigned by Blast2GO (https://www.blast2go.com/). *A*. *stenosperma* putative Transcription Factors (TFs) were identified from the functional BLASTX annotation, and classified according to their TF family using KEGG (http://www.genome.jp/kegg/).

For the MapMan ontology, all *A*. *stenosperma* DEGs were compared to *Glycine max* Affymetrix chip (http://soybase.org/AffyChip/), using BLASTN (E-values ≤1e-7) default parameters. Only *A*. *stenosperma* DEGs with identity hits higher than 70% were used for the analysis, and to compile a MapMan ontology (http://mapman.gabipd.org/) classification. Pictorial representations for the biotic stress pathways were uploaded from the MapMan website.

### Expression analysis by qRT-PCR

For qRT-PCR analysis, the Platinum^®^ SYBR^®^ Green qPCR Super Mix-UDG w/ROX kit (Invitrogen, USA) was used according to manufacturer's recommendations on ABI 7300 Real-Time PCR System (Applied Biosystem Foster City, USA). *A*. *stenosperma* RNA samples were reassembled in pools of five random individuals with two independent biological replicates formed per collecting point (control and 3, 6 and 9 DAI). A total of 2 μg of each RNA pool was treated with 2 U of DNase (Fermentas, Germany) and reverse transcribed using the Super Script II enzyme and oligo(dT) 20 primer (Invitrogen, USA), according to manufacturer’s instructions. Specific primer pairs were designed for the selected candidate genes (S2 Table) and PCR reactions carried out as previously described [14]. Average cycle threshold (Cq) values were estimated using the online real-time PCR Miner tool [19] and normalized to two reference genes (*As*60S and *As*GAPDH), as previously established [18]. Expression ratios of mRNA transcripts at 3, 6 and 9 DAI, relative to control were determined and statistically tested using REST 2009 ver. 2.0.13 software [20]. For qRT-PCR analysis using contrasting genotypes, a new bioassay was conducted in greenhouse conditions essentially as described above using *M*. *arenaria* resistant *A*. *stenosperma* (V10309) and susceptible *A*. *hypogaea* cv Runner. Total RNA from control and 6DAI inoculated roots were assembled in pools of three individuals to form two biological replicates per treatment.

## Results and Discussion

### RNA-sequencing and *de novo* assembly

Non-inoculated and *M*. *arenaria* race 1 inoculated (3, 6, 9 DAI) *A*. *stenosperma* roots were collected for total RNA extraction. Two biological replicates per collecting point were subsequently formed by pooling total RNA samples. The eight cDNA libraries constructed were pair-ended sequenced using Illumina Hi-Seq 2000, with two technical replicates for each library, in two different sequencing runs. A total of 141.3 million raw reads was produced, constituting 28.2 GB of cDNA sequences. An overview on the data of the RNA-seq experiment and contig mapping in the best *de novo* assembly is shown in Table 1 and S1 Fig. This Transcriptome Shotgun Assembly project (TSA) has been deposited at DDBJ/EMBL/GenBank under the accession GDBK00000000 (PRJNA284674). The version described in this paper is the first version, GDBK01000000.

**Table 1:** Summary of the *A*. *stenosperma* Illumina Hi-Seq sequencing data.

Library	Total number reads	≥ Q30^a^(%)a	Raw reads (%)	Number contigs	Mapped Reads (%)
**3 DAI—1**	23,015,612	93.9	16.2	41,523	35.0
**3 DAI—2**	15,466,334	94.5	10.9	40,648	39.9
**6 DAI—1**	14,229,647	95.3	10.3	36,749	36.0
**6 DAI—2**	20,684,933	95.6	14.6	37,926	35.6
**9 DAI—1**	15,294,055	95.0	10.8	37,061	39.0
**9 DAI—2**	16,126,462	95.2	11.4	37,077	37.7
**CTR—1**	15,994,785	94.6	11.3		36.6
**CTR—2**	20,517,124	95.1	14.5	8,581	35.5
**Total**	141,328,952				

^a^(Q30) 1 in 10^3^ error in base caling

The *de novo* transcript assembly consisted of 37,882 loci which include a combined total of 44,132 contigs (S1 Table). In total 3,548 of the loci had multiple contigs consisting of different combinations of splice variants or different regions of the same loci due to partial assembly. Most contigs were 200–300 bp in length, with more than 37,000 contigs common among all libraries (S1 Fig). Overall, 34,489 sequences (78.14%) showed similarity to proteins in the NCBI databank using BLASTX algorithm (E value < e-7) (S1 Table). The number of loci assembled for *A*. *stenosperma* with gene structure found herein, is comparable to those described in other tropical legumes such as soybean (43,367) [21], chickpea (42,141) [22], pigeonpea (48,680) [23] and common bean (39,572) [24].

To date, publicly available transcriptome data for *A*. *stenosperma* comprise 6,264 ESTs and 7,723 contigs originated from a Transcriptome Shotgun Assembly (TSA) both produced by this group [25, 26]. In this study, the sequencing of root samples during the early stages of the *A*. *stenosperma*/RKN interaction, associated to the robustness of the Illumina Hi-Seq technique, enabled a 3-fold increase on the number of loci identified for this species, which is known to harbor resistance to a number of pests but lacks a reference genome sequenced. This study will, therefore, expand considerably the transcriptomic resources available to be used for gene discovery, molecular genetics and functional genomics in the genus *Arachis*.

### Functional annotation

**Fig 1:**
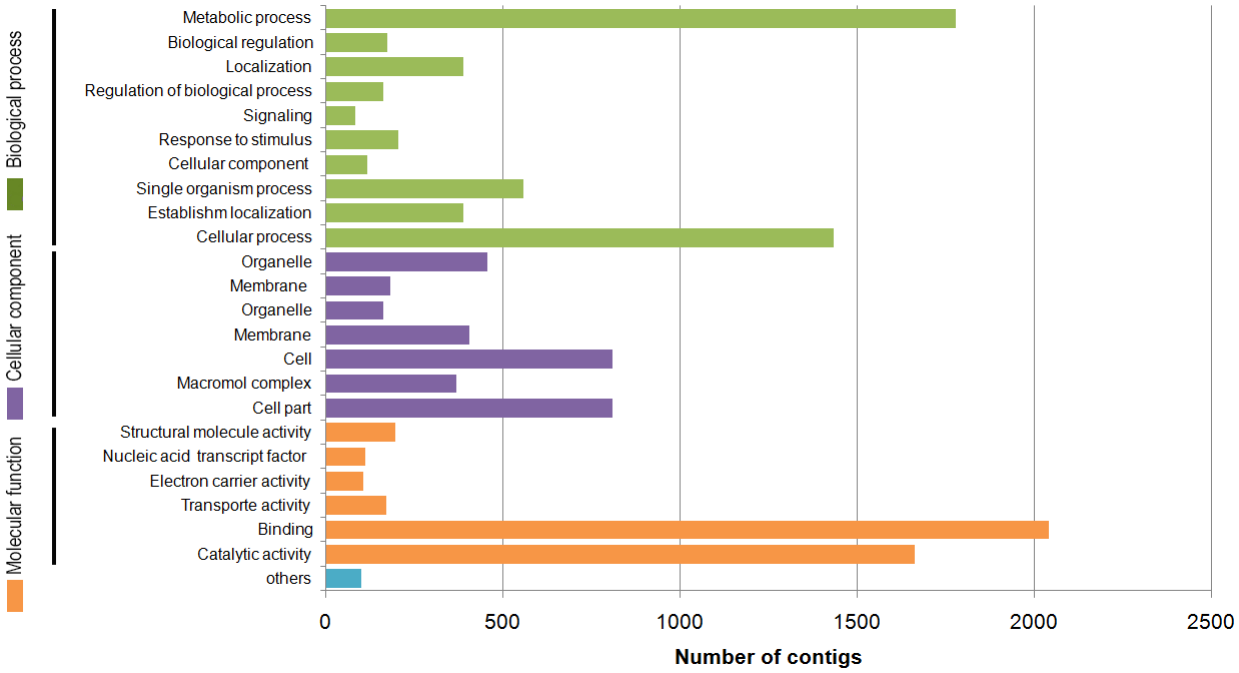
Gene Ontology (GO) terms of *A*. *stenosperma* transcriptome during the interaction with *M*. *arenaria*.

The annotation of the *A*. *stenosperma* contigs and their assignment into functional categories based on conserved PFAM domain predictions and gene ontology (GO) allowed the classification of almost 30% (13,157) of the 44,132 contigs in the three main GO categories: biological process (41.59%), molecular function (33.74%) and cellular component (24.66%) (Fig 1). This distribution was in accordance with previous analysis on peanut and wild *Arachis* transcriptome [16, 26–28], with proteins related to metabolic processes (15%) and cellular processes (12%) enriched in the biological process, whilst cell (8%) and cell parts (7%) were the most highly represented in the cellular components category. Interestingly, within the molecular function category, binding proteins (14%) and proteins related to catalytic activity (11%) were the most enriched, with only 207 contigs annotated in the response to stimulus category (0.5%) (Fig 1). This included genes with putative defense function regulated during RKN infection, such as peroxidases, chitinases, extensins and proteinase inhibitors, perhaps playing a global response to pathogen invasion or specific response to the nematode, including serine–threonine protein kinases and glycosyltransferases [29]. Genes related to regulation of hormones, such as auxin repressed proteins (ARP) and cytokinin oxidase, previously related to *A*. *stenosperma* response to *M*. *arenaria* were also identified [14, 30].

### Transcription factors distribution

**Fig 2:**
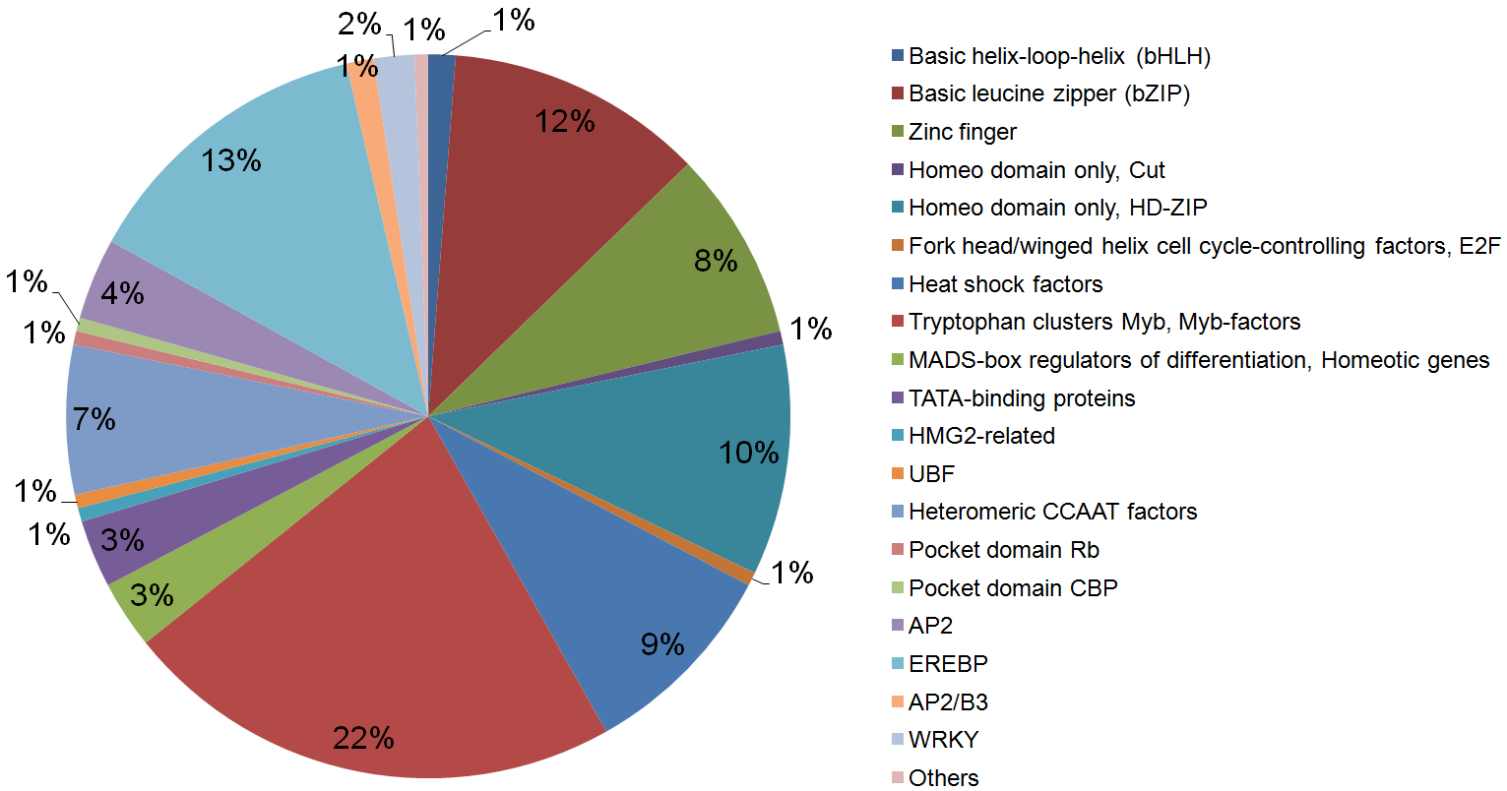
Distribution of *A*. *stenosperma* Transcription Factors families during the interaction with *M*. *arenaria*.

Transcriptional factors (TFs) are of foremost importance, as gene induction by stress occurs primarily at the level of transcription. TFs regulation of the temporal and spatial expression of these genes is an important part of downstream signaling pathways to initiate protective stress responses, as seen in peanut and other legumes [31–33]. *A*. *stenosperma* TFs here identified were classified into 23 families according to KEGG, and those related to biotic stress response were amongst the most abundant, including MYB (22%), EREBP (13%), bZIP (12%), HD-ZIP (10%), Heat shock (9%), Zinc finger (8%), CCAAT binding (7%) and WRKY (2%) (Fig 2).

The three most expressed TF families in this study MYB, EREBP and bZIP, which are implicated in many cell physiological and biochemical processes, have also been described as being involved in different plant-pathogen interactions, through the regulation of pathogen resistance and cell death [33]. Thus, it is possible that the TFs highly expressed here have closely overlapping functions, and might activate or repress genes through cis-acting sequences that respond to the nematode infection.

### Clustering analyses of the differentially expressed genes

Differential expression of *A*. *stenosperma* contigs was achieved with DESeq package that performs variance estimation and differential expression of the raw read counts, using the contig count matrix built from the Bedtools coverage output. Therefore, at each sampling point, the number of differentially expressed genes (DEGs) in the infected libraries was compared to the control (Fold Change-FC). Genes were considered to be differentially expressed when relative gene expression levels showed at least 4-FC difference (log_2_FC ≥2.0 or ≤ -2.0) between inoculated and control samples, with and P-value<0.05. In total, 958 (2.17%) significant DEGs were identified among the 44,132 contigs in at least one of the sampling points (3, 6 or 9 DAI), indicating that, the great majority of contigs were evenly expressed between inoculated and control roots during the assay time course.

**Fig 3:**
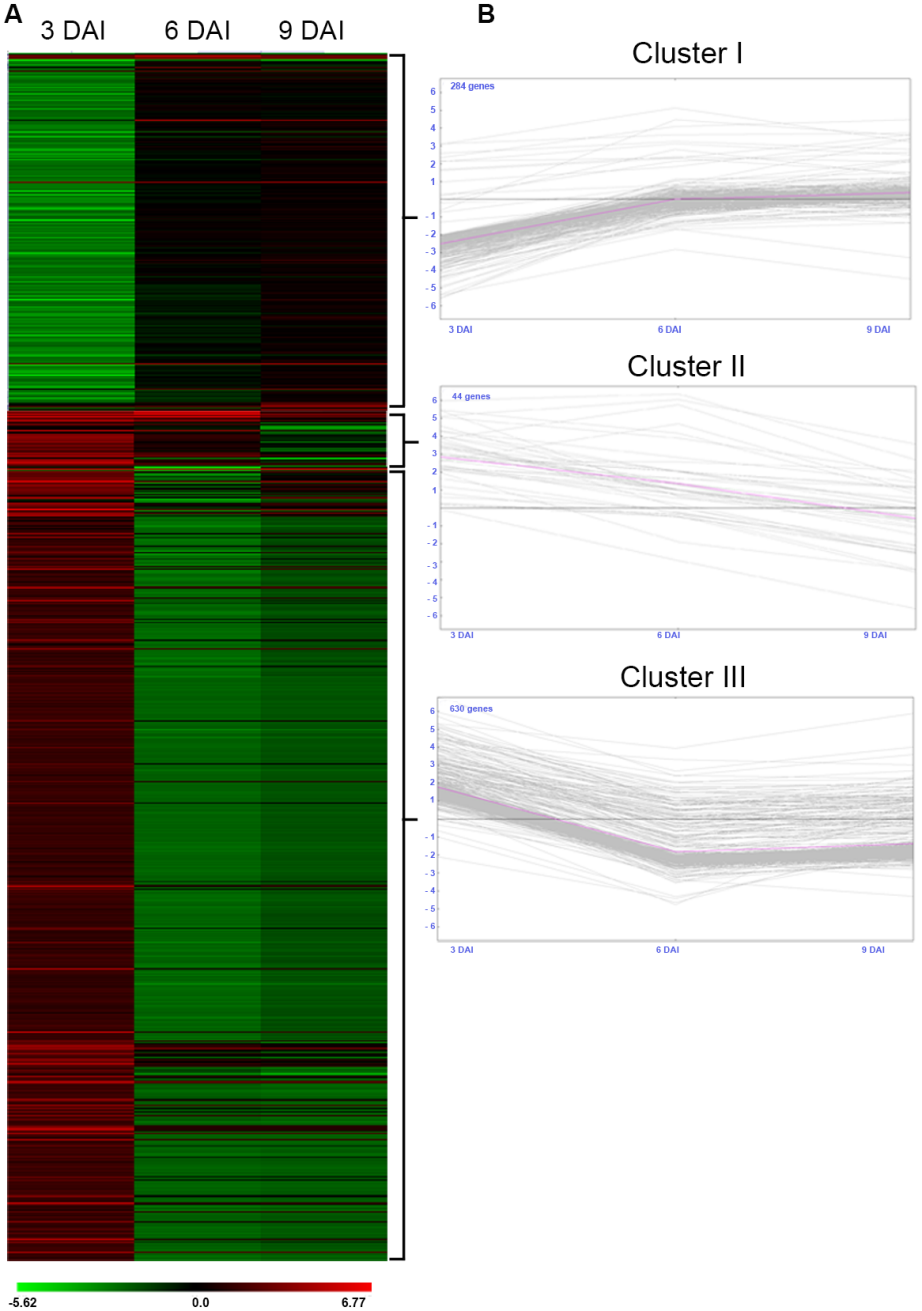
HeatMap of *A*. *stenosperma* Differentially Expressed Genes (DEGs). A) Distribution of *A*. *stenosperma* DEGs significantly (FC ≥2.0 or ≤ -2.0 and p˂0.05) up and downregulated during *M*. *arenaria* infection with in at least one of the sampling points (3, 6 or 9 DAI). Normalised values, relative to control, are shown in a red–green scale; the brightest red being the most upregulated and the brightest green, the most downregulated genes; B) DEGs K-means clustering analysis, with the pink lines indicating the representative expression profile of the Cluster; x- axes represent days after nematode inoculation (DAI) and y-axes, log2FC of mRNA levels of DEGs in the sampling points (3, 6 or 9 DAI) normalized to control.

A Heat Map representation of the clustering analysis of the global expression of these DEGs indicated a general downregulation during the initial time points of the *M*. *arenaria* infection (Fig 3A). Numbers of DEGs greatly differed at each stage of this plant-pathogen interaction and point up to a considerable imbalance of gene expression during nematode infection. At 3 DAI, the majority of DEGs (268) were dowregulated in *A*. *stenosperma* infected roots, whilst 165 were upregulated. The profile changed more drastically at 6 DAI, when the number of downregulated DEGs (473) is 52-fold higher than that of upregulated genes (9). At subsequent stage (9 DAI), most of the genes (91) was still repressed, and only few (13) were induced. This global gene downregulation at early stages of RKN interaction is in agreement with the expression profiles previously reported for peanut and *A*. *stenosperma* [30, 34], as well as for other legumes.

The expression trends at the early stages of *M*. *arenaria/A*. *stenosperma* interaction were corroborated by subsequent MeV clustering analysis (P< 0.05 and log_2_FC ≥2.0 or ≤ -2.0), which gathered the 958 significant DEGs into three distinct clusters based on common expression patterns (Fig 3A and 3B). Cluster I grouped 284 DEGs downregulated at 3 DAI, with a slight upregulation at 6 and 9 DAI (Fig 3B). Contrastingly, Cluster II showed 44 DEGs which were induced only at 3 DAI, followed by a steady downregulation at the two other points. The larger group, Cluster III, was composed of 630 DEGs displaying an upregulation only at 3 DAI, followed by a strong downregulation at 6 and 9 DAI. Representatives of each cluster were selected for further validation of their expression profile using qRT-PCR.

Regardless the trend of the expression pattern (up or down), the great majority of genes showed differential regulation at 6 DAI (482), followed by 3 DAI (433) and 9 DAI (104), with 61 showing significant regulation in more than one sampling point. Most commonly regulated genes were identified between 6 and 9 DAI, with few in common to 3 DAI, as most DEGs were downregulated at 6 and 9 DAI, but upregulated at 3DAI (Fig 3A and 3B).

Several studies, including those in *Arachis*, have identified genes responsive to nematode infection, in both, the compatible and incompatible interactions, which regulation vary dramatically due to, among other factors, the time elapsed since pathogen perception [30, 34 35]. In the resistant *A*. *stenosperma*, the great majority of genes are repressed in response to the infection, which is in accordance to studies on other plant-RKN interactions [35–37], and on the soybean cyst nematode [38]. This suggests that the downregulation of a significant number of genes might be necessary for the onset of the specific HR in *A*. *stenosperma*, as in other resistant genotypes, which is the main mechanism of resistance to *M*. *arenaria* [10, 39].

### MapMan ontology

**Fig 4:**
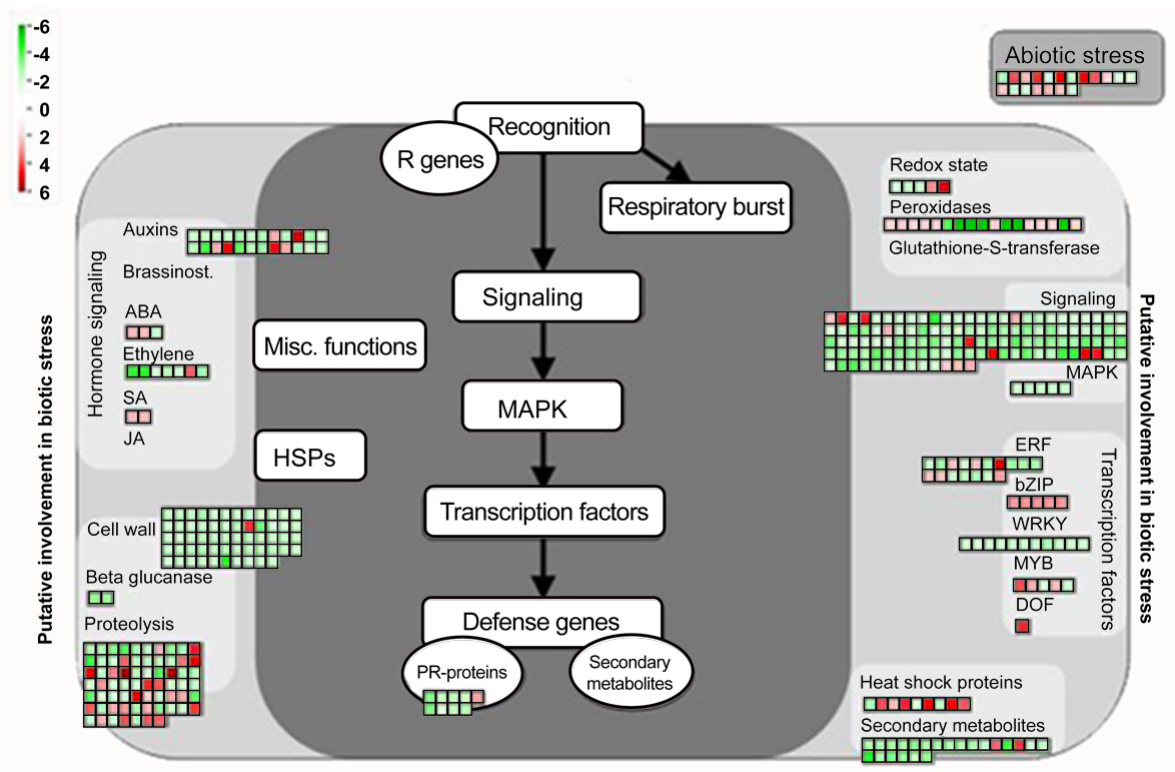
MapMan visualization of *A*. *stenosperma* genes involved in the response to *M*. *arenaria* infection. Overview of DEGs expression patterns (log_2_FC of mRNA levels) in infected roots relative to control. Dots show the different paralogous genes encoding proteins that are related to a certain defense response step. Red dots indicate upregulation and green dots downregulation.

In order to obtain an overview of the metabolic pathways or other processes underlined by the 958 DEGs, we used the MapMan tool [40]. For that, due to the lack of an Affymetrix chip containing *Arachis* spp. sequences, only *A*. *stenosperma* DEGs displaying sequence identity higher than 70% with the *G*. *max* Affymetrix chip sequences were considered for the analysis. A total of 405 genes (42.27%) with sufficient homology to the *G*. *max* genome probes were annotated into 20 MapMan Bins. A diagram displaying the expression of these DEGs into biotic stress pathways and processes is shown in Fig 4. Using MapMan annotation software to organize and display our data sets in the context of biological pathways we found 345 genes related to biotic stress responses among distinct DEGs, with a large number of genes involved in signaling (114), including 39 TFs. Interestingly, those TF genes revealed in MapMan analysis belong to TF families, as MYB, EREBP and b-ZIP (Fig 4), that are also the most abundant families previously identified in the TF distribuition analysis (Fig 2). Also, genes related to pathogen recognition and defense response (9), cell wall modification (58), hormonal metabolism (36) and proteolysis (67), enzymes of the phenylpropanoid pathway and others related to secondary metabolites (22), which are well known to be involved in the defense response to nematode infection, could be mapped (Fig 4).

Overall, MapMan ontology enabled a genome-wide expression outline of *A*. *stenosperma* genes responding to *M*. *arenaria* infection, by identifying pathways that are inlvolved in the main steps leading to the HR response. Innitially, pathogen recognition by R genes (Fig 4) triggers Effector-Triggered Immunity (ETI), eliciting reactive oxygen intermediates and ion fluxes (redox state species and peroxidases) and also enzymes that protect the cell from the attack by reactive oxygen (e.g. Glutathione-S-transferase). Following, the upregulation of genes engaged in signal transduction pathways (e.g. ERF, b-ZIP, MYB) promote the induction of a large set of genes commonly known as defense related genes (e.g. Heat Shock Proteins, Proteolitic proteins), Pathogenesis related proteins (PR) and enzymes involved in the synthesis of anti-microbial compounds called phytoalexins (secondary metabolites). Genes involved in phytohormone signaling pathway directly related to plant defense response (Jasmonic acid (JA), Salycilic Acid (SA)) and others that play a role in plant imunity (ABA and Auxin) were also upregulated (Fig 4). Some of the proteins and transcription factors regulated here are also known to be activated by environmental factors (abiotic stress), whilst PAMP-triggered immunity (PTI) activated by pathogen associated molecular patterns (PAMPs), activate cell wall enzymes, induce proteolysis and transcriptional reprogramming as a common plant immune responses (Fig 4).

### Genes expression profiling

From the 958 DEGs identified, representatives of each cluster (5%) identified in MeV cluster (Fig 2) were selected as candidate genes for expression profile validation by qRT-PCR. Within each cluster, candidate genes were chosen based on their fold-change level (FC), (positive- or negatively regulated), assuming that those with the highest fold-change ratio would be more likely to have a biological role in this plant-nematode interaction, and also based on their homology in BLASTX to proteins with a functional role in plant defense mechanisms. From those, 27 candidate genes showed significant (P<0.05) differencial expression in at least on time point by RT-qPCR analysis (Table 2 and S2 Table). Among these candidates, representatives of different steps of the plant defense response, previously identified in MapMan analysis (Fig 4), were found, including: (i) genes associated to the perception of PAMPs such as glucan binding proteins (*AsBger*) that perceives beta-glucans from microbes; (ii) enzymes that degrade pathogen cell wall such as chitinases (*AsCHI2*); (iii) resistance genes such as NBS-LRR proteins (*AsTIR-NBS-LRR*) that recognize pathogen effectors and (iv) TFs previously identified *in silico* as the most abundant and related to plant responses to stresses (*AsMYB25*; *AsWRKY49*; *AsERF6*) (Fig 2). In addition, genes codifying for (v) key enzymes on the jasmonic and salicylic acids metabolic pathways, which are triggered as plant responses to pathogen attack, such as ureases (*AsUreD*) and Response Regulator (*AsPRR37*), (vi) enzymes related to Reactive Oxygen Response (ROS), such as allene oxidase (*AsAOC3*) and (vii) enzymes related to hormonal balance (*AsGH3*.*1*) and (ix) cell wall composition and trafficking, such as two cell wall hydrolases (*AsCWAH* and *AsCWAH2*), were also found to be significantly differentially regulated.

**Table 2:** Differentially expressed candidate genes in *A*. *stenosperma* roots infected with *M*.*arenaria*.

Gene symbol	Acc ID	Putative gene (BlastX)	Related taxon	E-value
*AsALKBH2*	A_stenosperma_Nema_Hiseq_L_29780_T_1	Alpha-Ketoglutarate-dependent dioxygenase Homolog 2-like	*Cicer arietinum*	1,00E-91
*AsAOC3*	A_stenosperma_Nema_Hiseq_L_517_T_1	Allene Oxide Cyclase 4	*Glycine max*	2,00E-130
*AsAraH8*	A_stenosperma_Nema_Hiseq_L_35385_T_1	Ara h 8 allergen	*Arachis hypogaea*	3,00E-10
*AsATPase α*	A_stenosperma_Nema_Hiseq_L_1605_T_2	ATP synthase subunit alpha	*Medicago truncatula*	2,00E-22
*AsAUX/IAA*	A_stenosperma_Nema_Hiseq_L_13_T_1	Auxin-Responsive protein	*Phaseolus vulgaris*	1,00E-78
*AsBap*	A_stenosperma_Nema_Hiseq_L_33039_T_1	Beta-adaptin-like protein A-like	*Glycine max*	9,00E-51
*AsBger*	A_stenosperma_Nema_Hiseq_L_24150_T_1	Beta-glucan-elicitor receptor	*Glycine max*	1,00E-105
*AsBTB*	A_stenosperma_Nema_Hiseq_L_26421_T_1	BTB/POZ domain-containing protein	*Camelina sativa*	2,00E-29
*AsCHI2*	A_stenosperma_Nema_Hiseq_L_30251_T_1	Class II Chitinase	*Arachis hypogaea*	4,00E-133
*AsCOX1*	A_stenosperma_Nema_Hiseq_L_3048_T_7	Cytochrome c oxidase subunit 1	*Millettia pinnata*	0.0
*AsCWAH*	A_stenosperma_Nema_Hiseq_L_36188_T_1	Cell wall-associated hydrolase	*Rothia dentocariosa M567*	7,00E-49
*AsCWAH2*	A_stenosperma_Nema_Hiseq_L_32291_T_1	Cell wall-associated hydrolase	*Medicago truncatula*	1,00E-10
*AsERF6*	A_stenosperma_Nema_Hiseq_L_14392_T_1	Ethylene-Responsive element binding Factor 6	*Arachis hypogaea*	7,00E-100
*AsGH3*.*1*	A_stenosperma_Nema_Hiseq_L_5661_T_1	Probable indole-3-acetic acid-amido synthetase GH3.1	*Glycine max*	0.0
*AsIOMT*	A_stenosperma_Nema_Hiseq_L_11970_T_1	Isoflavone 7-O-methyltransferase	*Glycyrrhiza echinata*	8,00E-149
*AsKEL*	A_stenosperma_Nema_Hiseq_L_37157_T_1	Kelch repeat-containing protein-like	*Arachis hypogaea*	2,00E-56
*AsMAN*	A_stenosperma_Nema_Hiseq_L_18389_T_1	Mannan endo-1,4-beta-mannosidase 2-like	*Glycine max*	1,00E-62
*AsMYB25*	A_stenosperma_Nema_Hiseq_L_5798_T_1	MYB-related protein 25	*Arachis hypogaea*	4,00E-44
*AsNDX1*	A_stenosperma_Nema_Hiseq_L_29095_T_1	NDX1 homeobox protein	*Medicago truncatula*	2,00E-10
*AsPRR37*	A_stenosperma_Nema_Hiseq_L_934_T_1	Response regulator-like APRR7-like	*Cicer arietinum*	1,00E-24
*AsSAG*	A_stenosperma_Nema_Hiseq_L_31635_T_1	Senescence-Associated protein	*Medicago truncatula*	3,00E-31
*AsSLP*	A_stenosperma_Nema_Hiseq_L_34699_T_1	Subtilisin-like protease-like	*Glycine max*	4,00E-44
*AsTAT*	A_stenosperma_Nema_Hiseq_L_15416_T_1	Tyrosine aminotransferase	*Medicago truncatula*	4,00E-163
*AsTIR-NBS-LRR*	A_stenosperma_Nema_Hiseq_L_10567_T_1	TIR NB-ARC LRR protein	*Arachis duranensis*	4,00E-103
*AsTMV*	A_stenosperma_Nema_Hiseq_L_19034_T_1	TMV resistance protein	*Glycine max*	2,00E-39
*AsUreD*	A_stenosperma_Nema_Hiseq_L_27820_T_1	Urease accessory protein UreD	*Medicago truncatula*	2,00E-122
*AsWRKY49*	A_stenosperma_Nema_Hiseq_L_3478_T_2	WRKY49	*Glycine max*	5,00E-56

**Fig 5:**
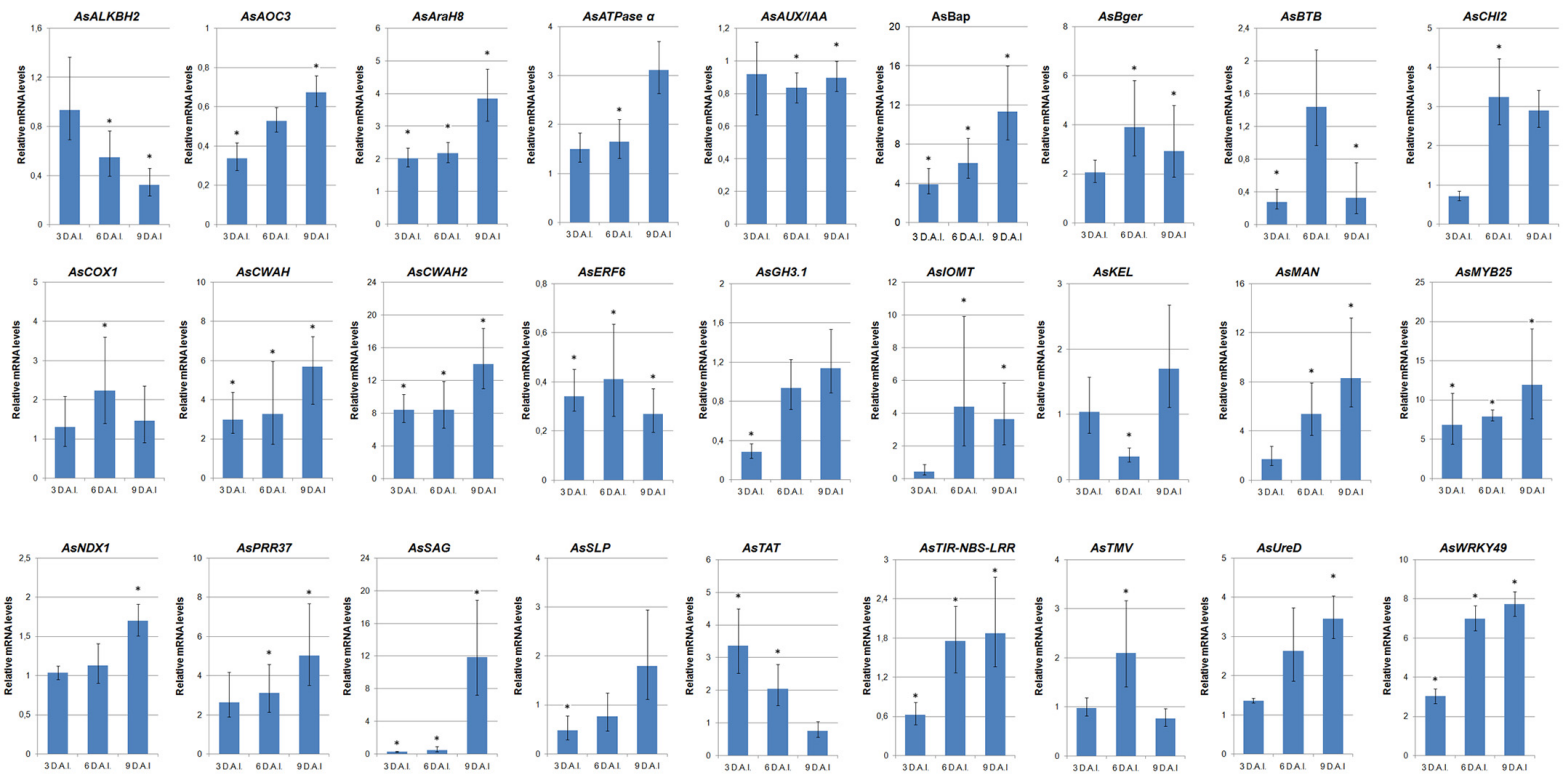
*A*. *stenosperma* candidate genes expression profile by qRT-PCR. Relative mRNA levels of 27 candidate genes in *A*. *stenosperma* roots inoculated with *M*. *arenaria* collected at 3, 6 and 9 days (DAI), relative to control. Bars represent the standard deviation of two biological replicates. Significantly (P < 0.05) up- or downregulated genes are indicated *.

All 27 candidate genes analyzed by qRT-PCR showed specificity of transcript amplification, with high amplification efficiencies (S2 Table). Their mRNA levels in *A*. *stenosperma* roots inoculated with *M*. *arenaria* relative to control are shown in Fig 5. The majority of candidate genes (17) displayed a gradual increase in their expression as the nematode infection progressed, achieving a positive regulation at 9 DAI (Fig 5). *AsAOC*, showed an unique profile, as it also increased its expression along the assay but was still negatively regulated at 9 DAI. Their expression profile corresponded to genes previously classified in Cluster I (Fig 3). The opposite expression profile, consisting of a gradual decrease on gene expression along the assay, was observed only for two genes (*AsALKBH2* and *AsTAT*; Fig 5) representatives of Clusters II (Fig 3). For the remaining eight candidate genes (*AsAUX/IAA*, *AsBerg*, *AsBTB*, *AsCHI2*, *AsCOX1*, *AsERF6*, *AsKEL* and *AsTMV*), 6 DAI was the inflection point of the curve representing their expression profiles (U- and V-shaped) (Fig 5), with (*AsAUX/IAA* and *AsKEL*) being representatives of Cluster III.

**Fig 6:**
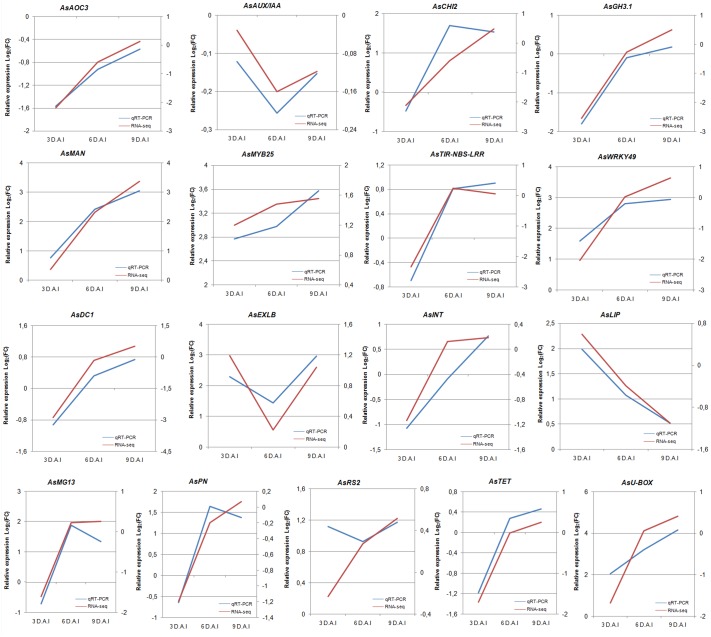
Comparisons between RNA-seq and qRT–PCR expression profile. Log2 transformed relative mRNA levels of DEGs in roots of *A*. *stenosperma* inoculated with *M*. *arenaria* collected at 3, 6 and 9 DAI relative to control, analysed by qRT-PCR (left Y-axis) and by RNA-Seq right Y-axis.

As a follow-up analysis, the expression profiles of these candidate genes by qRT-PCR and *in silico* were compared. For that, graphs displaying the transcript abundance for each contig inferred *in silico* (log2 transformed relative mRNA levels) and the relative quantification obtained for the corresponding transcript by qRT-PCR (log2 transformed) were built (Fig 6). An additional set of nine genes previously identified in our studies as interesting candidates for nematode resistance [14, 26] was also included to broaden the analysis (*AsMG13*, *AsU-BOX*, *AsLIP*, *AsDC1*, *AsPN*, *AsINT*, *AsTET*, *AsRS2* and *AsEXLB*) (S3 Table). When primers designed for these additional candidate genes [14] (S3 Table) amplified by electronic PCR more than one contig in our RNA-Seq database, only the contig with the expression profile most similar to the qRT-PCR was considered (Fig 6).

Overall, 17 DEGs showed a comparable expression profile between both analyses, with qRT-PCR results showing to be positively correlated to RNA-Seq data analysis (Pearson correlation coefficients r = 0.68). This set of genes exhibited distinctive responses to nematode challenge, and include those related to phytohormones signaling, TFs, cell wall structure and trafficking, which participate in plant resistance to nematodes, including the onset of the HR response, the resistance mechanism described for *A*. *stenosperma* against *M*. *arenaria* [10].

The association of their expression profiles in response to nematode infection with their primary functions in defense might contribute to unravel their putative roles during this incompatible plant-nematode interaction, as described below.

#### Genes involved in the salycilic acid pathway

After perception of pathogen or microbe–associated molecular patterns (PAMPS), which are sensed through leucine-rich repeat (LRR) receptors, plants induce stomatal closure and produce innate immunity responses. If the plant has a specific resistance gene (R) that recognizes the corresponding avirulence (*avr*) gene from the pathogen, a rapid defense mechanism known as the HR occurs to prevent infection. In general, salycilic acid (SA) activates the defense response to biotrophic and hemi-biotrophic pathogens, induces Systemic Acquired Resistance (SAR), and triggers the expression of SAR-associated pathogenesis related genes (PRs). SA has already been described as playing an important role in decreasing susceptibility to RKN, as it can cause accumulation of Reactive Oxygen Species (ROS), which is observed during the early stages of incompatible interactions and it is one of the components of the HR reaction [41–43].

From the relatively few endoparasite nematode resistance (Nem-R) genes isolated so far, the vast majority fall into the NBS-LRR class of R-genes, including the *MI* gene from tomato, which confers resistance to *M*. *incognita* and *M*. *javanica* and five *Me* genes in pepper, that control different species of *Meloidogyne* [39, 44, 45]. In the *A*. *stenosperma/ M*. *arenaria* interaction, several typical cell features, including densely stained cytoplasm and altered organelle structures were observed in the central cylinder, indicating a R-gene mediated HR of infested host cells [10]. In the present study, two members of the NBS-LRR class of R-genes, *AsTIR-NBS-LRR* and *AsMG13* showed an increased level of expression in *A*. *stenosperma* roots, in concert with the onset of the HR response, what suggests their involvement in the early stages of defense response in this incompatible interaction (Fig 6). R-genes might detect directly the pathogen avirulence effector or recognize changes in the host protein elicited by the pathogen effector (guard hypothesis) [46], and can be used in combinations to be deployed in cultivars to maximize their durability against pathogen changes. The identification of new distinct R genes is, therefore, an important topic in most crop improvement programmes, and *AsTIR-NBS-LRR* and *AsMG13* constitute, thus, interesting candidate genes for further functional characterization.

High levels of SA that often lead to HR are regulated by other proteins, such as lipocalins, which are involved in modulating tolerance to oxidative stress [47]. These proteins have already shown to act as scavengers of potentially harmful molecules known to be induced by extreme temperature stress and excessive light [48]. In our study, the lipocalin *AsLIP* gene expression is induced after nematode perception (3 DAI) following by a sharp reduction on its expression along the assay (Fig 6), which might contribute to the outbreak of the HR in this species. In our previous studies, another known ROS plant scavenger, a metallothionein homolog (*AsMET*) also had its expression diminished during the *A*. *stenosperma/M*. *arenaria* interaction [30], reinforcing the potential role of these molecules in this defense mechanism.

Salicylic acid is a known secondary metabolism inducing agent, including the phenylpropanoid pathway that, on its turn, produces a variety of structural and defense-related phenolics including resveratrol [49]. Resveratrol Synthase (RS) mediates the synthesis of resveratrol and is induced by different stresses, [48] constituting an important regulator for initiation of HR-related cell death [50]. Peanut is one of the few crop plants that is able to synthesize resveratrol and the accumulation of RS and its mRNA has been previously observed in wild *Arachis* in response to treatment with ultraviolet light [51]. In our previous studies [14], the RS homolog *AsRS2*, showed upregulation along the nematode infection (3, 6 and 9 DAI), whilst here, similar trend was observed *in silico* at at 6 and 9 DAI (Fig 6), both coinciding with the occurrence of HR in *A*. *stenosperma*. Together with other genes involved in the SA pathway described here, *AsRS2* is suggested to be involved in the HR response of *A*. *stenosperma* to *M*. *arenaria*.

In addition to this immediate, gene-a-gene contest, plants also employ general resistance mechanisms induced after the HR to combat secondary infections from a broad spectrum of pathogens, known as systemic acquired resistance (SAR). This mechanism is commonly associated with activation of genes that code for defense related proteins, including chitinases, that have been shown to degrade chitinous components of eggs of *M*. *incognita* causing a significant reduction on their development and reproduction rate [52]. Here, we found a chitinase homolog (*AsCHI2*) upregulated at 6 and 9 DAI (Fig 6), which we suggest as having a part on the SAR response of *A*. *stenosperma* to the pathogen.

#### Genes involved in the jasmonic acid pathway

Besides the genetic resistance mediated by R-genes, induced resistance (IR), activated under the influence of metabolites of plant pathogens and various biotic and abiotic factors called elicitors also occurs. Jasmonic acid (JA) and ethylene are important for the induction of nonspecific disease resistance, and play an important role in IR activating genes that are involved in plant defense in response to wounding and pathogen attack [53]. Jasmonates are closely associated with the production of secondary metabolites, such as flavonoids, and can also trigger synthesis of a wide range of defensive proteins in roots, which are toxic to nematodes *in vitro*, and have been implicated in plant defense against these parasites [54]. One of these compounds are Patatin-like proteins, that play an essential role in HR and cell death during plant-pathogen interactions, as these proteins mediate the production of JA and defense responses in some species [55]. Previous works have shown the upregulation of patatin in response to *M*. *arenaria* infection in *Arachis* spp. [14, 34]. Here, the induction of *AsPN* was confirmed both *in silico* and in RT-qPCR (Fig 6), at 6 and 9 DAI, the time points corresponding to the induction and aftermath of the HR in *A*. *stenosperma*, which reinforces its role in this defense response.

Another protein involved in mounting resistance responses via the accumulation of JA is allene oxidase cyclase (*AsAOC3*), which has been demonstrated to be a functional enzyme involved in the biosynthesis of JA and related compounds [56]. In this study, *AsAOC3* showed a good correlation between the *in silico* and qRT-PCR expression profiles (Fig 6). However, its role in this plant-nematode interaction is not yet clear, because regardless showing a trend of increased expression along the whole assay, this candidate gene was downregulated at all time points analysed (Fig 6).

#### Genes related to hormonal balance, cellular plasticity and signaling

Auxin signaling, which is generally recognized to be involved in plant growth and development, is also involved in the complicated network of plant–nematode interactions, with evidences that RKN interfere in auxin biosynthesis in feeding cells, thus, altering local hormone balance in order to form the giant cell complex [57]. Here, an Auxin Responsive Protein (*AsAUX/IAA*) was downregulated in response to *M*. *incognita* infection, in both qRT-PCR and *in silico* analysis, especially at 6 DAI (Fig 6). Moreover, previous analyses by macroarray and Northern blotting, showed that *AsARP*, a gene repressed by the presence of auxin, was strongly upregulated in *A*. *stenosperma* roots infected with *M*. *arenaria* [30]. These findings, the downregulation of an auxin-induced gene, *AsAUX/IAA*, and the upregulation of an auxin-repressed gene, *AsARP*, corroborate our previous hypothesis that the lack of auxin accumulation could lead to a failure in giant cell formation in incompatible host-RKN interactions, being part of the suite of defense genes regulated by hormones harbored by *A*. *stenosperma*.

Another constituent of the complex network of auxin signaling that regulates stress adaptation responses in plants is GH3), which encodes an IAA-amido synthetase that maintains auxin homeostasis by conjugating excess IAA (indole-3-acetic acid) to amino acids [58]. GH3 also inhibits expansin production, which on its turn is induced by IAA [59]. Expansins are cell wall-associated proteins that contribute for the cell wall plasticity and may also contribute to the plant vulnerability to pathogens. Therefore, the suppression of expansin might contribute to plant resistance to some pathogens. Considering that GH3 is induced by multiple pathogens, its mediated role in nematode resistance might be due, at least partly, to inhibiting the expression of expansins by suppressing auxin signaling [59].

In our study, *AsGH3*.*1*, which is an enzyme that prevents the free accumulation of auxin, showed a steady increase on its expression immediately after nematode perception (3 to 6 DAI) (Fig 6). At the same interaction period, an expansin homolog (*AsEXLB*) displayed a notable decrease on its expression, specially at 6 DAI, when HR is initially observed in the *A*.*stenosperma/M*.*arenaria* interaction [10] (Fig 6). This antagonist gene expression between *GH3*.*1* and expansin homologs has previously been described in rice infected with *Xanthomonas oryzae*, and proved to be a mechanism which contributed to an increased resistance to the pathogen [58]. In this study, we suggest that *AsGH3*.*1* and *AsEXLB* may also be interligated and contribute to the host defense response. Therefore, we hypothesize that *M*. *arenaria* initially (3 DAI) induces auxin production by the plant, in order to weaken the cell wall as an attempt to establish the giant cell [35]. However, the resistant *A*. *stenosperma* in turn, defends itself from auxin-facilitated nematode infection by preventing the loosening of its cell wall via suppressing auxin accumulation, as indicated by *AsAUX/IAA* and *AsARP* expression behavior discussed above. This is through the activation of *AsGH3*.*1* by the pathogen that, in its turn, inactivates IAA, by conjugating it to amino acids, preventing cell wall-loosening and nematode penetration, and therefore the establishment of the giant cell by the nematode.

To further support the role of membrane structural proteins in *A*. *stenosperma/M*.*arenaria* interaction, two other membrane-associated genes, coding for Tetraspanin (*AsTET*) and Integrin (*AsINT*) also displayed a compatible profile of increased expression in both, *in silico* and qRT-PCR (Fig 6). Tetraspanin is a membrane spanning protein containing a large extracellular loop, which provides interactive binding sites and reportedly has a role in development, pathogenesis and immune response in animal and plants [60]. The main suggested function for Tetraspanin is cell-to-cell signaling, which is also one of the main roles attributed to Integrins, that play a critical part in cell structure, migration and anchoring cells to the extracellular matrices [61]. A well known Integrin-like protein is the non-race specific disease resistance NDR1 gene in plants, a plasma membrane-localized protein that plays an essential role in resistance mediated by the NBS-LRR proteins, as it is a key signaling component of these responses during pathogen infection [62]. In our study, both *AsTET* and *AsINT* showed a steady increase in their expression along plant-nematode interaction, with upregulation after 6 DAI (Fig 6). This contributes to the assumption of their role in *A*. *stenosperma* in cell signaling and triggering the plant defense response against nematode parasitism.

Besides structural proteins, cell wall enzymes are thought to be important components of the mechanisms by which plants sense pathogens, including endo-ß-mannanases [63], which overexpression has shown to increase resistance to root fungus in transgenic tobacco [64]. Here, *AsMAN* was upregulated during the whole period of the nematode-plant interaction analysed (Fig 6). Altough it is well known that carbohydrate moieties on nematode surface are specific elicitors, functioning in the determinative stages of nematode-plant interactions [65], no indicative of mannan eliciting resistance by RKN has yet being described.

#### Transcription factors and DNA binding elements

The responses to pathogen invasion require large-scale transcriptional reprogramming, including those in some TF families. In this study, two defense related TFs, *AsMYB25* and *AsWRKY49*, were shown to be upregulated in response to infection in both *in silico* and RT-qPCR analyses (Fig 6). These candidates are members of MYB and WRKY TF families identified as some of the most abundant in this study (Fig 2), suggesting their putative role in this incompatible plant-nematode interaction. Here, *AsWRKY49* increased its expression along the assay (Fig 6) and might be involved in the host response either by directly increasing the expression of PRs, or through the mediation of the NPR1 protein [66]. Likewise, MYB genes have been identified in other plant species displaying resistance to endosedentary nematodes [67], which contributes to the assumption that some representatives of this TF family in *A*. *stenosperma* may be involved in the response to *M*. *arenaria* parasitism.

Additionally, DC1 domain-containing zinc finger proteins have been demonstrated to play an important role in regulating the defense responses to biotic stresses, as they function as positive regulators of plant cell death programme (PCD) and SA-dependent defense responses [68]. In this study, an *A*. *stenosperma* DC1 homolog, *AsDC1* is induced at 6 and 9 DAI in response to the nematode infection both *in silico* and in qRT-PCR. Comparably, similar expression trend was observed for another protein containing a *AsU-box*, that through ubiquitin ligase activity, may act as regulators in defense against pathogen and PCD in plants [69].

#### Expression profiling of candidate genes in resistant and susceptible *Arachis* genotypes

In order to verify whether the 17 candidate genes selected above (DEGs) (Fig 6) are involved in global responses to RKN infestation in any *Arachis* species or in specific responses by RKN-resistant *A*. *stenosperma*, their expression profiles were analysed in two contrasting *Arachis* genotypes for *M*. *arenaria* resistance: *A*. *stenosperma* (resistant) and *A*. *hypogaea* cv Runner (susceptible). For that, qRT-PCR analysis was conducted with samples from a new bioassay including control and inoculated roots of the two contrasting *Arachis* genotypes, using primers designed for *A*. *stenosperma* (S2 Table).

**Fig 7:**
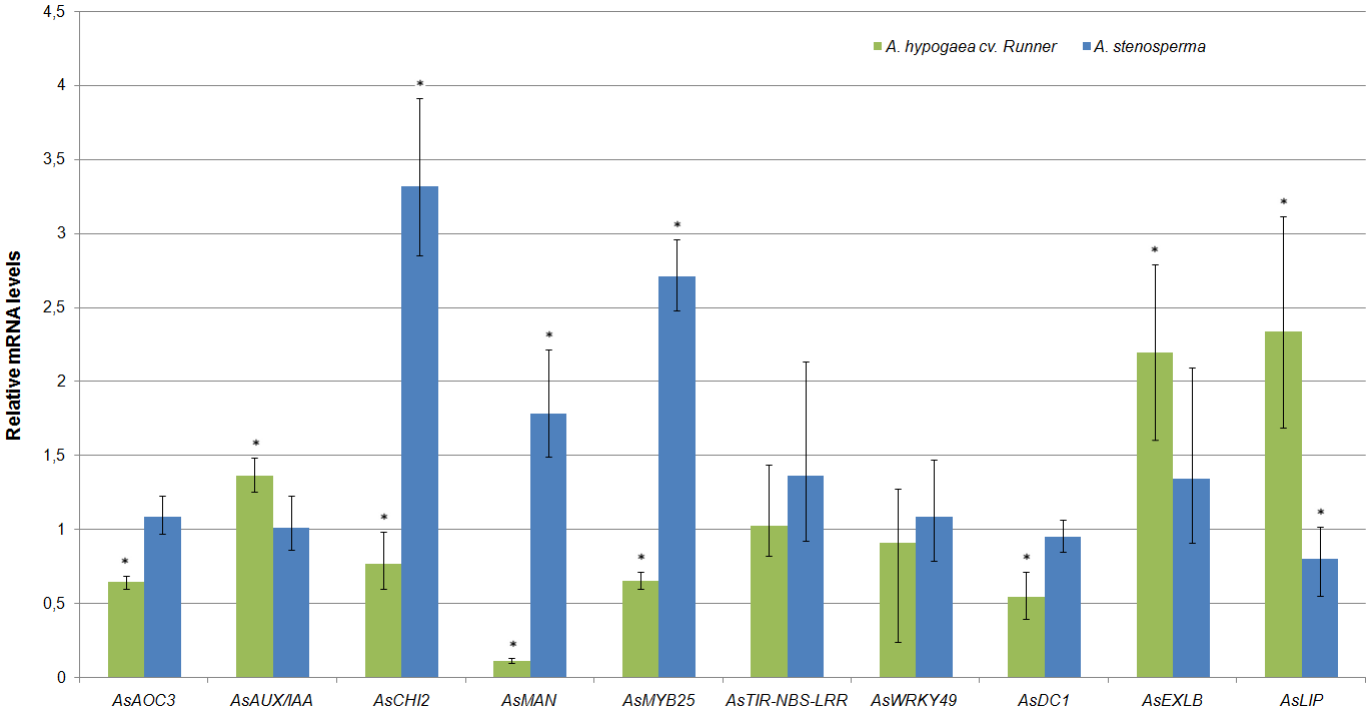
*A*. *stenosperma* and *A*. *hypogaea* qRT–PCR expression profile. Relative mRNA levels of 10 candidate genes in *A*. *stenosperma* and *A*. *hypogaea* roots inoculated with *M*. *arenaria* relative to control. Bars represent the standard deviation of two biological replicates. Significantly (P < 0.05) up- or downregulated genes are indicated by*.

For 10 DEGs analyzed, single peaked melting curves were generated, indicating a specific amplicon in both *A*. *hypogaea* and *A*. *stenosperma*. Contrasting expression behaviors (up or downregulation) between the two genotypes were observed for eight genes (*AsOAC*, *AsAUX/IAA*, *AsCHI2*, *AsMAN*, *AsMYB25*, *AsTIR-NBS-LRR*, *AsWRKY49* and *AsLIP*) (Fig 7). The two remaining DEGs (*AsDC1* and *AsEXLB*), whereas showing a similar response to RKN infection (downregulation for *AsDC1* and upregulatuion for *AsEXLB*), differed considerably in their fold change magnitude in each genotype (Fig 7).

The discongruity in expression behavior of DEGs representing PRs, ETI receptors, and jasmonic acid (JA)- and salicylic acid (SA)-related proteins in RKN-resistant and susceptible genotypes following *M*. *arenaria* inoculation, coupled with their putative role in HR response discussed throghout this study, reinforce their involvement in the specific responses by RKN-resistant *A*. *stenosperma*, as in contrast to transcripts involved in global responses to RKN infestation in other genotypes.

Current knowledge of signaling mechanisms that operate during plant infestation by parasitic nematodes are limited, especially in wild species. In the present work, a deep transcriptome analysis allowed the identification of differentially expressed genes during the early stages (3 to 9 DAI) of the incompatible *A*. *stenosperma*/*M*. *areanaria* interaction. These genes, either inlvolved in the PAMP-triggered immunity (PTI) or Effector-Triggered Immunity (ETI), were further validated by qRT-PCR, corroborating their potential as genes of interest. We found that both JA-dependent (patatin, allene oxidase cyclase) and SA-dependent (R genes, lipocalin, resveratrol synthase, chitinase) defense genes and their regulators (MYB, WRKY) were triggered in *A*. *stenosperma* roots when infected with *M*. *arenaria*. Additionaly, proteins that are involved in the regulation of defense responses (DC1, U-BOX), cell signaling (mannanases, tetraspanin, integrin), cell plasticity (expansin) and hormonal balance (auxin responsive, GH3) were also differentially expressed in infected roots, with a number of them showing divergent expression profile in contrasting genotypes for *M*. *arenaria* resistance, reinforcing their role in specific responses by RKN-resistant *A*. *stenosperma*. Most of these genes constitute interesting candidates for further functional analysis, and their knockdown or gain-of-function effect in *A*. *rhizogenes*-induced hairy roots in composite peanut plants [70], will contribute to decipher their potential role in *A*. *stenosperma* defense mechanisms, including HR and SAR against *M*. *arenaria*.

The fact that genes participating in both defense hormone pathways (SA and JA) were identified in this plant-nematode interaction probably indicates the absence of a conflict between these signaling systems, as observed in the *Mi* mediated resistance in tomato [71] and other plant species [72, 73], where cross-talk between different hormone pathways has been shown to optimize the defense response against antagonists. These findings encourage the use of different signaling pathways mediating defense in peanut plants to optimize their defensive responses with both genetic (HR) and induced resistance (IR), which can be used in single or cumulative properties, and contribute to the reduction of its susceptibility to nematodes and possibly, also to other pests. Pyramiding or alternating resistance genes against *Meloidogyne* spp. has already proved to be effective in other crops [45], and once used to produce improved peanut cultivars, may also contribute to a more durable resistance and improve the long-term control of root-knot nematodes in the species.

## Conclusions

Parasitic RKN evolved sophisticated strategies to infect plant causing worldwide yield losses. Sources of host resistance against the most damaging nematode species in peanut (*M*. *arenaria*) have been identified in its wild relative *A*. *stenosperma* and is due to the onset of the Hypersensitive Response. The present study suggests putative genes that trigger changes in specific signalling pathways either inlvolved in the PAMP-triggered immunity (PTI) or Effector-Triggered Immunity (ETI), and also in structural arrangements involved in *A*. *stenosperma* resistance to RKN. The deployment of individual or combined resistance genes by marker assisted selection or via transgenic expression can offer several benefits for nematode control in an integrated management strategy, reducing risks to the environment and to human health, accessibility for food, and the possibility of achieving durable, broad-spectrum nematode resistance.

## Supporting Information

S1 Table *A*. *stenosperma* contigs and their best protein homology at NCBI (BLASTX). (XLSX)

S2 Table Primer sequences of the differentially expressed genes (DEGs). (DOCX)

S3 Table Additional differentially expressed candidate genes in *A*. *stenosperma* roots infected with *M*. *arenaria*. (DOCX)

S1 Fig *A*.*stenosperma* ILLUMINA HI-Seq 2000 statistics. (TIF)
