# Supplementary material for: Root Transcriptome Analysis of Wild Peanut Reveals Candidate Genes for Nematode Resistance
Source: PLoS One. 2015 Oct 21;10(10):e0140937. doi: 10.1371/journal.pone.0140937 (PMC4619257; doi:10.1371/journal.pone.0140937)
Supplement: S2 Table — (DOCX) [file pone.0140937.s002.docx]

S2 Table- Primer sequences of the differentially expressed genes (DEGs)

| Gene symbol | amplicon size (bp) | Amplification efficiency ± s.d. | Primer sequence | | |
| --- | --- | --- | --- | --- | --- |
|  |  |  | Forward (5'-3') | Reverse (5'-3') | |
| *AsALKBH2* | 155 | 1.008 ± 0.008 | GAAGCAGCGAACCCTAATGA | GTGGGTCTGGTCCATGAGAT | |
| *AsAOC3* | 187 | 1.047 ± 0.005 | TCATGTCAACGTAGCAGAATCC | CTAAGGCCTGTCACCCTCAT | |
| *AsAraH8* | 92 | 1.035 ± 0.012 | TCACAGTCATGGCGGTCTTCAC | GGAGTCGGCATCCTTCAAAGCA | |
| *AsATPase α* | 185 | 1.136 ± 0.015 | TCCTGTTTGCTCCCCACGCTTT | TGTGAAATCCCCGGGCTCAA | |
| *AsAUX/IAA* | 150 | 0.842 ± 0.006 | AAATGGCCTCTTCAGCTTCA | CCAGTTGCCTCAGAACCTTT | |
| *AsBap* | 100 | 0.873 ± 0.002 | TCGGGTGGCAAACTTGGTGGAA | CACAACCCCAATAACTGCCACA | |
| *AsBger* | 167 | 1.023 ± 0.006 | ACAAACCCGAGGGCAAACACGT | AGAGAATGGCGTGGATGGTGGT | |
| *AsBTB* | 159 | 1.057 ± 0.013 | TGTGCAGAACCATGCTTGCCCT | CTAAGGGTTTCGTTGGGTTTCAGT | |
| *AsCHI2* | 103 | 1.046 ± 0.007 | TGCTGAATCACGTGTCTCACCA | AGCAGGGCAAGCATTGTCATCT | |
| *AsCOX1* | 151 | 1.016 ± 0.007 | GCAGTCGGCGACCTTTTCTTCT | AGGCGAACATTCCTTGGGCTGT | |
| *AsCWAH* | 152 | 1.028 ± 0.008 | ACGTAGCCAATCAGCCATGCAC | AGCTGGGTTCAGAACGTCGTGA | |
| *AsCWAH2* | 109 | 1.112 ± 0.010 | TCAAAAGGCACGCCGTCACA | CGTGAGGGAATGGTGAAAAGCAC | |
| *AsERF6* | 119 | 1.114 ± 0.019 | TCATGCACCCTTTCTTCGAACCC | GTCTGTGGCTGAGACACTGCAA | |
| *AsGH3.1* | 156 | 1.102 ± 0.013 | TGGCCAAGAGACGTTCCAGT | TTGGGCCTACCGAGTTTAAGTCT | |
| *AsIOMT* | 152 | 1.057 ± 0.008 | TGCCTTCTTTGACTCTCTTTGCC | GCATTAAGCGATGCACACCACT | |
| *AsKEL* | 189 | 1.076 ± 0.014 | TCAAGTGTTCACGCAGGCATGA | TCATCCTTTTGGTCACTGGGTGC | |
| *AsMAN* | 121 | 1.003 ± 0.009 | TGCTGACTATCGGTCATGAAGGT | TTCGAGATGTTGGAGTTCCGGA | |
| *AsMYB25* | 150 | 1.057 ± 0.008 | CAAAGACCACCTCTCACCAACC | ATAATGGGTGACGCGGATCGA | |
| *AsNDX1* | 106 | 1.051 ± 0.007 | TGGGGGAGGCTCATCACCTTTA | GCTGGTCTATGCCTTGGAATGC | |
| *AsPRR37* | 159 | 0.879 ± 0.018 | TCCACACACAAACACCGCGCAA | ACCGTGGAGAACCAAACCTCCA | |
| *AsSAG* | 110 | 1.02 ± 0.007 | AAGCCCGTTCCCTTGGCTGTTA | AGGTAGCCAAATGCCTCGTCA | |
| *AsSLP* | 148 | 1.082 ± 0.010 | TCACTGGAGTCCCGCTGCTATT | TGCAAGGTTTGGTCGAACATGT | |
| *AsTAT* | 178 | 1.035 ± 0.005 | AAGATGTGGCCCTTGACCCTGT | GCCACACTGCAATGAGGATCTC | |
| *AsTIR-NBS-LRR* | 174 | 1.104 ±0.012 | AGCATTTAACTGCAGCGCGTGA | TGTCTTGCTTGTACACCCACCG | |
| *AsTMV* | 164 | 1.075 ± 0.010 | ACGCAAAGAGCATTGTCGGGGA | AGGGGCACAAGCGTGTTACTCA | |
| *AsUreD* | 199 | 1.044 ± 0.008 | CCTTCAGGTGCCTTGAGTCACA | AAGGTGGCACCCCAAGCAATGA | |
| *AsWRKY49* | 164 | 1.029 ± 0.008 | GGCCAGAAGGACAATCACCCTT | AGGTTCCTCCTTGGCTCTGGAA | |
| *AsMG13, AsU-BOX, AsLIP, AsDC1, AsPN, AsINT, AsTET, AsRS2 primers were according to Morgante et al. (2013)* | | | | | |
| *AsEXLB* primers were according to Brasileiro et al (2015) | | | | | |
