# Supplementary material for: Root Transcriptome Analysis of Wild Peanut Reveals Candidate Genes for Nematode Resistance
Source: PLoS One. 2015 Oct 21;10(10):e0140937. doi: 10.1371/journal.pone.0140937 (PMC4619257; doi:10.1371/journal.pone.0140937)
Supplement: S3 Table — (DOCX) [file pone.0140937.s003.docx]

| **Gene symbol** | **Acc ID** | **Putative gene (BlastX)** | **Related taxon** | **E-value** | **Reference** |
| --- | --- | --- | --- | --- | --- |
|  |  |  |  |  |  |
| *AsDC1* | A_stenosperma_Nema_HiSeq_L_1436_T_3 (EH044012) | DC1 domain-containing protein | *Medicago truncatula* | 2,00E^-34^ | Morgante al. (2013) |
| *AsEXLB* | A_stenosperma_Nema_HiSeq_L_23298_T_1 (JR342047) | Expansin like B protein | *Medicago truncatula* | 2,00E^-144^ | Guimarães et al (2012) |
| *AsINT* | A_stenosperma_Nema_HiSeq_L_510_T_1 (JR330501) | Integrin like | *Cicer arietinum* | 2,00E^-164^ | Morgante al. (2013) |
| *AsLIP* | A_stenosperma_Nema_HiSeq_L_3764_T_1 (EH042075) | Lipocalin | *Solanum lycopersicum* | 1,00E^-112^ | Morgante al. (2013) |
| *AsMG13* | A_stenosperma_Nema_HiSeq_L_2700_T_2 (EH047440) | Resistance protein MG13 | *Glycine max* | 2,00E^-54^ | Morgante al. (2013) |
| *AsPN* | A_stenosperma_Nema_HiSeq_L_1112_T_4 (EH047773) | Patatin-like protein | *Gossypium hirsutum* | 1,00E^-99^ | Morgante al. (2013) |
| *AsRS2* | A_stenosperma_Nema_HiSeq_L_6409_T_6 (EH048133) | Resveratrol synthase | *Arachis hypogaea* | 0.0 | Morgante al. (2013) |
| *AsTET* | A_stenosperma_Nema_HiSeq_L_2227_T_1 (GW276025) | Tetraspanin-LEL-like | *Glycine max* | 3,00E^-114^ | Tirumalaraju et al. (2011) |
| *AsU-BOX* | A_stenosperma_Nema_HiSeq_L_7769_T_1 (EH047058) | Ubiquitin-protein ligase | *Glycine max* | 0.0 | Morgante al. (2013) |

**S3 Table** – Additional differentially expressed candidate genes in *A. stenosperma* roots infected with *M. arenaria*
