# Supplementary figures and images for: Root Transcriptome Analysis of Wild Peanut Reveals Candidate Genes for Nematode Resistance
Source: PLoS One. 2015 Oct 21;10(10):e0140937. doi: 10.1371/journal.pone.0140937 (PMC4619257; doi:10.1371/journal.pone.0140937)

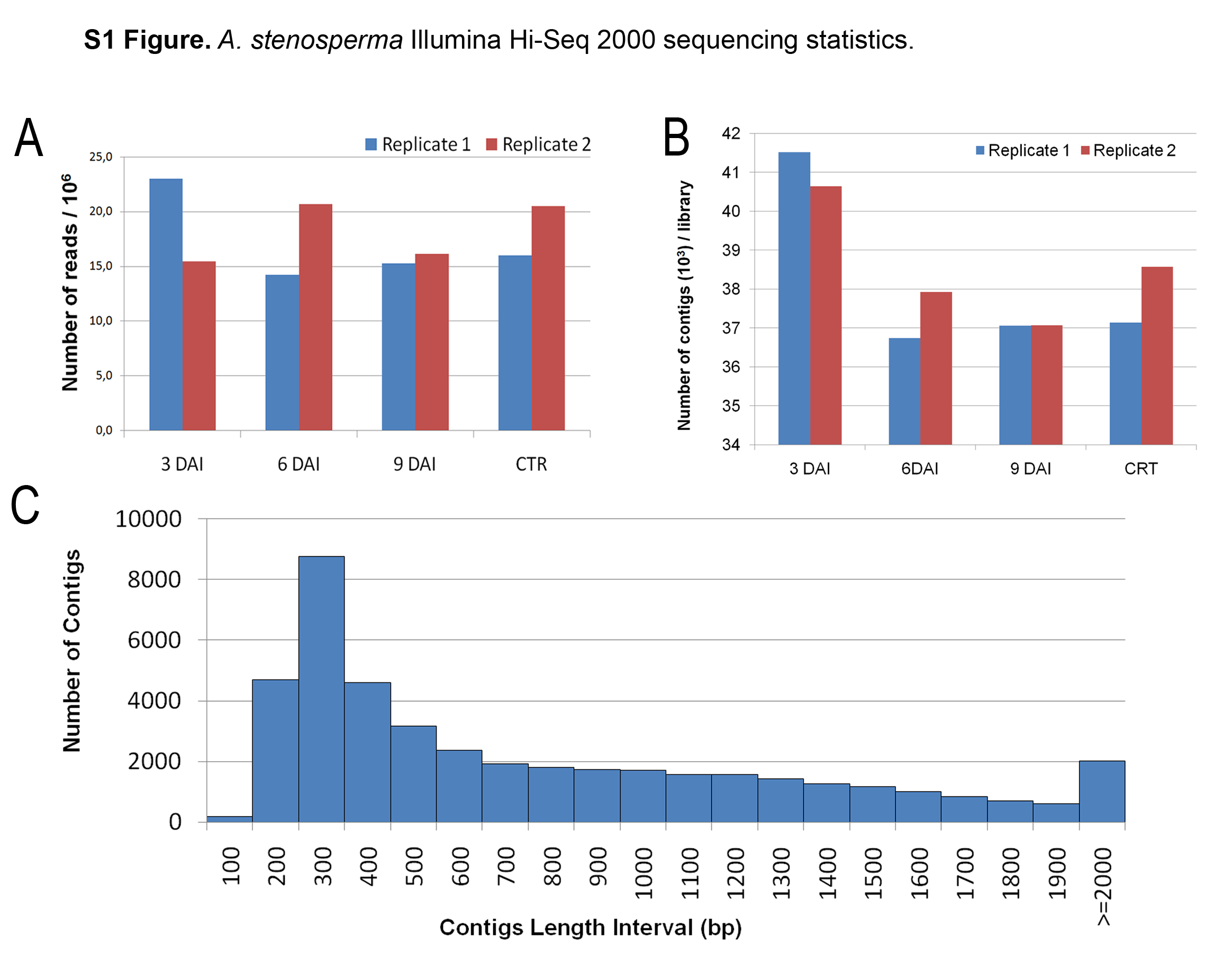

Supplement: S1 Fig — (TIF) [file pone.0140937.s004.tif]
